# Erythropoietin and its receptors in the brainstem of adults with fatal falciparum malaria

**DOI:** 10.1186/1475-2875-8-261

**Published:** 2009-11-22

**Authors:** Isabelle M Medana, Nicholas PJ Day, Tran Tinh Hien, Nicholas J White, Gareth DH Turner

**Affiliations:** 1 Nuffield Department of Clinical Laboratory Sciences, The John Radcliffe Hospital, University of Oxford, Oxford, UK; 2 Centre for Clinical Vaccinology and Tropical Medicine, Churchill Hospital, Oxford, UK; 3 Mahidol-Oxford Research Unit, Bangkok, Thailand; 4 Hospital for Tropical Diseases, Ho Chi Minh City, Viet Nam

## Abstract

**Background:**

Facilitation of endogenous neuroprotective pathways, such as the erythropoietin (Epo) pathway, has been proposed as adjuvant treatment strategies in cerebral malaria. Whether different endogenous protein expression levels of Epo or differences in the abundance of its receptor components could account for the extent of structural neuropathological changes or neurological complications in adults with severe malaria was investigated.

**Methods:**

High sensitivity immunohistochemistry was used to assess the frequency, distribution and concordance of Epo and components of its homodimeric and heteromeric receptors, Epo receptor and CD131, within the brainstem of adults who died of severe malaria. The following relationships with Epo and its receptor components were also defined: (i) sequestration and indicators of hypoxia; (ii) vascular damage in the form of plasma protein leakage and haemorrhage; (iii) clinical complications and neuropathological features of severe malaria disease. Brainstems of patients dying in the UK from unrelated non-infectious causes were examined for comparison.

**Results:**

The incidence of endogenous Epo in parenchymal brain cells did not greatly differ between severe malaria and non-neurological UK controls at the time of death. However, EpoR and CD131 labelling of neurons was greater in severe malaria compared with non-neurological controls (*P *= .009). EpoR labelling of vessels was positively correlated with admission peripheral parasite count (*P *= .01) and cerebral sequestration (*P *< .0001). There was a strong negative correlation between arterial oxygen saturation and EpoR labelling of glia (*P *= .001). There were no significant correlations with indicators of vascular damage, neuronal chromatolysis, axonal swelling or vital organ failure.

**Conclusion:**

Cells within the brainstem of severe malaria patients showed protein expression of Epo and its receptor components. However, the incidence of endogeneous expression did not reflect protection from vascular or neuronal injury, and/or clinical manifestations, such as coma. These findings do not provide support for Epo as an adjuvant neuroprotective agent in adults with severe malaria.

## Background

The ability of the brain to adapt to a range of insults may be critical in determining whether patients are protected from neurological complications and death during severe malaria infection. Boosting endogenous protective mechanisms is a potential treatment strategy of current interest in neurological diseases [1]. Erythropoietin (Epo) is a haematopoietic growth factor produced primarily in the adult kidney. Epo and its receptors are also expressed in tissue outside the haematopoietic system and Epo has been identified as a cytoprotective agent in both neuronal and vascular systems. Administration of exogenous Epo is neuroprotective in models of ischaemic and metabolic stress but may aggravate neuronal damage when administered during transient hypoxia [2]. Whether Epo is indifferent, protects or damages the brain appears to be related to the amount of Epo reaching the brain (reviewed in [3]).

Coma is a strong predictor of fatal outcome in severe malaria across all age groups [4]. Neurological sequelae are rarely observed in south-east Asian adults recovering from cerebral malaria (CM), whereas a significant minority of African children suffer gross neurological sequelae and a greater proportion have evidence of long-term neurocognitive impairment (reviewed in [5]). High serum levels of Epo have been associated with a reduced risk of neurological sequelae in children with malaria in retrospective studies but cerebrospinal fluid (CSF) levels did not reflect protection [6]. These data, together with results from the *Plasmodium berghei *ANKA murine model have focused attention on Epo as a potential neuroprotective adjuvant therapy in CM [7-9]. Clinical trials of Epo are underway in stroke and in African children with cerebral malaria [10,11]

In this study, immunohistochemical techniques have been used to investigate endogenous levels of Epo and Epo receptor components in the medulla of the brainstem of cases of fatal severe malaria in Vietnamese adults. The brainstem was chosen for examination in this study as severe malaria is often associated with brainstem signs. The reticular activating system, involved in the maintenance of consciousness, lies within the core of the pons and medulla. Damage to the cardio-respiratory centres in the medulla will lead to death.

There are few studies detailing the expression of Epo and its receptors in non-tumour-related human brain disease in adults [12-14] and using sensitive detection systems so the first aim was to define the frequency and distribution of expression in the medulla of severe malaria and non-infectious deaths. Comparisons with control groups are important to ensure differentiation of features specific to malaria compared to background non-specific agonal neuropathology. Hypoxia induces Epo and Epo receptor expression [14-18]. Parasitized erythrocyte adherence to cerebrovascular endothelial cells, a process termed sequestration, causes microvascular obstruction. Combined with other systemic complications of severe disease, such as anaemia and shock, could cause hypoxic damage to the brain in severe malaria (reviewed in [19]). It is, therefore, important to define the relationship between sequestration, the expression of Epo and its receptors and indicators of hypoxia. Epo has been reported to inhibit blood-brain barrier (BBB) permeability [20], so the relationship between Epo and its receptors and leakage of plasma proteins into the brain parenchyma and frank vascular damage in the form of haemorrhage was also investigated.

Neurological complications reflect neuronal dysfunction and Epo and its derivatives have been shown to provide histological evidence of neuronal protection and clinical improvement in a range of animal models [21-23]. The relationship was investigated between endogenous Epo and its receptors, and potentially reversible neuronal injury in the form of chromatolysis [24] and axonal injury that are characteristic features of CM in southeast Asian adults [25,26]. Epo is primarily produced by the kidney and is induced in response to anaemia, so the impact of other systemic complications of malaria disease, including renal failure and anaemia, on Epo expression in the brain was investigated. Finally, although neuroprotective Epo signalling is not fully understood it has been hypothesized to occur through two receptors: a high affinity, homodimeric (EpoR/EpoR), and a low-affinity, heteromeric (CD131/EpoR) receptor (reviewed in [27]). CD131, also known as the common beta receptor, is a signal transducing subunit shared by the granulocyte-macrophage colony stimulating factor, and the interleukin (IL)-3 and IL-5 receptors [3,28]). The concordance and relative abundance of EpoR and CD131 in serial sections of medulla of the brainstem was determined and the relationship with structural brain changes was investigated.

## Methods

### Case selection

Autopsy brain specimens were taken within 24 hours of death from adult patients who had died of severe falciparum malaria on the Malaria Ward, Centre for Tropical Diseases, Ho Chi Minh City, Vietnam, as described previously [29]. These patients were divided into two groups: CM (n = 10) and non-CM (n = 10). CM was defined according to World Health Organization guidelines on the basis of a Glasgow coma score of 11 or less [30], other causes of unconsciousness having been excluded (e.g. hypoglycaemia, meningitis or other encephalopathy), by clinical, biochemical and CSF examination. Non-CM patients were those dying from severe malaria without coma, who had a range of clinical features typical of other vital organ system complications (see Table 1 for more details).

**Table 1:** Malaria patient data

***Patient no***	***Drug***	***Age (yrs)***	***Sex***	***Time to death (h)***	***GCS*** ***Admission → Death***	***Convulsions ***	***CSF opening pressure (mmHg)***	***Additional Clinical History***
CM1	Q	36	F	52	6 → 3	No	High (20)	J, A, ARF, HG, S
CM2	A	22	M	38.5	7 → 3	No	Normal (9.5)	J, HP, HG, S
CM3	A	69	M	336	8 → 3	No	ND	J, A, ARF, HG, S
CM4	Q	34	M	6.6	8 → 3	No	Normal (10)	J, A, ARF, PO, HG, S
CM5	Q	36	M	20.66	8 → 3	Yes	High (23)	HP, J, ARF, HG, S
CM6	Q	30	M	36	7 → 3	No	Normal (17)	J, A, ARF, PO, S
CM7	Q	29	M	9	6 → 3	No	High (24)	PO, S
CM8	Q	63	M	16	10 → 3	No	Normal (9)	A, HG, PO, S
CM9	Q	44	M	39	7 → 3	No	High (21)	J, ARF, HG
CM10	A	44	F	24	7 → 3	Yes	Normal (17)	A, S
								
NCM1	Q	22	M	6.33	14 → 3	No	ND	J, HP, PO, S
NCM2	Q	63	M	50	11 → 3	No	High (21.5)	J, A, ARF, PO, S
NCM3	Q	43	F	94.5	15 → 3	No	ND	HP, J, A, ARF, HG, S
NCM4	Q	25	M	124	15 → 3	No	ND	J, A, HG
NCM5	Q	22	M	6	11 → 3	No	Normal (14)	HP, J, A, ARF, S
NCM6	Q	22	F	27.3	12 → 3	No	ND	HP, J, ARF, HG, S
NCM7	Q	54	M	35	11 → 3	Yes	Normal (13.5)	J, A, ARF, S
NCM8	A	56	F	4.66	13 → 3	No	ND	J, ARF, S
NCM9	Q	24	M	113	14 → 3	No	ND	J, A, S
NCM10	A	50	F	264	15 → 3	No	High (19)	J, A, ARF, S

Abbreviations: Q, quinine; A, artemether; CM, cerebral malaria; NCM, non-cerebral severe malaria; J, jaundice; A, anaemia; ARF, acute renal failure; HP, hyperparasitaemia; HG, hypoglycaemia, ND, not determined; S, haemodynamic shock, PO, pulmonary oedema.

Control cases (n = 12) were from different causes of death in patients undergoing autopsies at the John Radcliffe Hospital, Oxford, UK [31]. Autopsy delays varied but were predominantly conducted within 24-48 hours of death. These cases were divided into neurological and non-neurological deaths (see Table 2). Protocols for tissue sampling, storage and use were approved by the Ethical and Scientific Committee of the Centre for Tropical Diseases, Vietnam, OXTREC 029-02, COREC (C01.002) and the Human tissue authority license number 12217.

**Table 2:** Control Patient data

***Patient ***	***Age (y)***	***Sex***	***Neurological history***	***Additional Clinical History/Cause of death***
C1	30	M	N/A	Congenital myelodysplasic syndrome; abscess.
C2	56	F	Stupor due to depression/schizophrenia	Dehydration; bronchopneumonia.
C3	35	M	N/A	Extensive severe burns of the skin; smoke inhalation.
C4	35	M	Epilepsy; previous encephalitis.	Pulmonary oedema
C5	27	M	Unconscious; right-sided focal fit.	Pulmonary oedema; hypoglycaemia.
C6	23	F	N/A	Sickle cell trait; severe haemorrhage.
C7	45	M	N/A	Pulmonary oedema; splenomegaly.
C8	39	F	N/A	Chronic renal failure; gastric bleed.
C9	43	F	Ischaemic brain injury from hypovolaemic C-R arrest.	Renal transplant; CMV hepatitis; C-R arrest.
C10	78	F	Confusion; hyperosmolar non-ketotic diabetic coma.	Rheumatoid arthritis; anaemia; heart failure; non-insulin-dependent diabetes mellitis; bronchopneumonia.
C11	73	M	Infarction with cerebral atrophy; impaired cerebral function; coma.	Mitral valve replacement; cardiac failure; renal failure.
C12	86	M	Infarction; brain atrophic; perivascular oedema.	Bilateral bronchopneumonia and pleural fibrosis; myocardial infarction.

Abbreviations: N/A, not applicable. No history of neurological signs or symptoms; C-R, cardiorespiratory; CMV, cytomegalovirus. C1-C8, Brains showed no structural histological abnormality on standard neuropathological examination.

### Immunohistochemistry

Immunolabelling was assessed either using a semi-quantitative scale evaluated at the microscope by two independent observers or by semi-automated, quantitative image analysis. Epo, EpoR and CD131 were preferentially assessed by eye using the semi-quantitative scale to allow the distinction of labelling phenotype by a range of different cell types and vessels of different sizes. The image analysis method evaluates the total amount of immunostaining over the section and is therefore more suitable for markers that give a diffuse staining pattern and are difficult to give a subjective score, such as fibrinogen.

Brain samples were collected at autopsy, preserved in 10% formalin for approximately six weeks, embedded in paraffin and sectioned on a microtome just prior to immunostaining. Control brain samples were acquired at a similar time period and paraffin tissue blocks were exposed to identical storage conditions as the malaria cases in the NDCLS, Oxford. Immunohistochemistry was performed on 5 μm paraffin-embedded tissue sections of medulla using the following antibodies or antisera: Epo (polyclonal, Abcam, Cambridge 1:65), CD131 (rabbit monoclonal, Abcam, 1:100), EpoR (monoclonal anti-human, R&D Systems Europe, Abingdon) and fibrinogen (rabbit antisera, DAKO, Ely). Paraffin sections were dewaxed, rehydrated and then underwent microwave antigen retrieval or proteinase K digestion (for fibrinogen only: 5 min, room temperature, DAKO, S3020).

Bound antibody was visualized using the EnVision HRP kit, Catalysed signal amplification kit (DAKO) or the Novolink Polymer detection system (Leica Biosystems Newcastle Ltd, UK). Positive controls in addition to fatal neurological deaths from the UK (Table 2) included sections of lymphoma as indicated by the antibody manufacturer. Negative controls comprised sections immunostained as above apart from omission of the primary antibody or replacing the primary antibody with an isotype control.

### Semi-quantitative scoring of immunostaining

The degree of Epo, EpoR or CD131 immunolabelling associated with vessels (small and large), glia and neurons was semi-quantitated using the following scale: no staining/grade 0, <1% cells or vessels staining/grade 1, 1 - 10% cells or vessels staining/grade 2, >10% cells or vessels staining/grade 3. The relative abundance of CD131 to EpoR was determined: 0, no staining for either CD131 or EpoR; 1, grade of immunolabelling for CD131 < EpoR; 2, grade of immunolabelling for CD131 = EpoR; 3, grade of immunolabelling for CD131 > EpoR.

### Histopathological correlation

Epo, EpoR and CD131 were visualized using exceptionally high sensitivity detection systems (Leica Microsystems, DAKO) that preclude the possibility of double labelling procedures on the same tissue sections. Identification of the cellular expression of these markers was based on nuclear morphological and dimensional characteristics as well as location within the gray and white matter of the medulla. Neurons were identified in the gray matter by their large nuclei and prominent nucleolus as well as pigment granules, Nissl's substance or lipofuscin within the cytoplasm. Glia can be subdivided into astrocytes, microglia, oligodendrocytes and ependymal cells but no attempt was made to distinguish between sub-types for this study. Glia were distinguished from neurons by nuclear phenotype. In general, glial nuclei are smaller, lack nucleoli but often contain clumps of heterochromatin and frequently are found in a satellite position. Blood vessels were identified by their narrow tubular profiles. Layers of the vessel walls can be clearly identified using counterstains with endothelial cells providing the inner most layer and smooth muscle cells, pericytes and perivascular macrophages in the surrounding layers.

Concordant or discordant expression of Epo, EpoR and CD131 were assessed by performing immunolabelling on serial sections. Since our semi-quantitative scoring system is based on the number (or incidence) of cells staining rather than the intensity of staining then concordant expression would only occur in serial sections with identical scores (although the reverse is not necessarily true).

### Quantitation of fibrinogen using digital image analysis

Fibrinogen immunolabelling was quantified using a modified version of a semi-automated method previously used for β-amyloid precursor protein in tissue sections from severe malaria cases [26]. Briefly, tissue sections were digitized using the EverSmart Pro II flat bed scanner (CreoScitex, Canada). Regions of low, medium and high levels of immunolabelling were selected by density thresholding of grey scale converted images using SigmaScan Pro5 image analysis software (SYSTAT, San Jose, CA). Thresholds were selected by comparison of the staining intensity on non-neurological and neurological UK control sections as guides for setting the computerized low and high thresholds respectively. These were kept constant between cases. The total area of the tissue sections was calculated. The amount of fibrinogen load was expressed as the area of tissue covered by staining divided by the total area of the section in square μm.

### Clinicopathological correlation

Correlations of immunohistochemical staining patterns for the various markers were conducted with clinical complications of severe malaria including: anaemia (haematocrit < 20% plus parasitaemia > 100 000 μl), haemodynamic shock (systolic blood pressure < 80 mmHg), pulmonary oedema, hypoglycaemia (plasma glucose < 2.2 mmol/L), admission peripheral parasitaemia, jaundice (bilirubin >2.5 mg/dL), hyperlactataemia (plasma lactate>5 mmol/L) and acute renal failure (plasma creatinine >3 mg/dL). All clinical and biochemical analyses were performed as part of the patients' clinical work-up during their stay in hospital. No analyses were performed on long-term, frozen archival plasma/CSF samples. Retrospective analysis of Epo protein in archival CSF samples from these patients was not performed since growth factors such as Epo only survive in frozen CSF for less than three months [32].

### Statistics

Data were analysed using non-parametric tests where appropriate (Kruskal-Wallis test, Spearman rank correlation, and Fisher's exact test) using SPSS 13 (Chicago, USA). No adjustments for multiple comparisons were made, although, for the purposes of interpretation and discussion, *P *< .01 was regarded as significant.

## Results

### Frequency and distribution of Epo, EpoR and CD131 immunolabelling

Few studies have analysed Epo, EpoR or CD131 on paraffin-embedded human brain tissue so each primary antibody was tested with several visualization systems. Optimal staining could only be obtained using exceptionally high sensitivity, polymer technology or catalysed signal amplification systems that are reported to have up to 50-fold greater sensitivity compared with traditional (strept)avidin-biotin complexes (DAKO). The down side of improved sensitivity was the preclusion of labelling multiple proteins on the same tissue section. Epo and EpoR were visualized using the Novolink Polymer detection system (Leica Biosystems Newcastle Ltd, UK) and CD131 was only detectable using the catalysed signal amplification kit (CSA, DAKO). It is unlikely that the low CD131 signal reflects degradation of the antigen in the malaria sections since the CSA kit was also required for optimal staining of the lymphoma positive control sections. Weak avidity of the antibody or low level protein expression in brain are in keeping with a recent study of CD131 in rat brain [33]. No differences were observed between the frequency of Epo or EpoR labelled cells visualized with the Novolink or CSA kit but high diffuse background noise was less frequently observed with Novolink for these antibodies.

#### Epo

**Figure 1:**
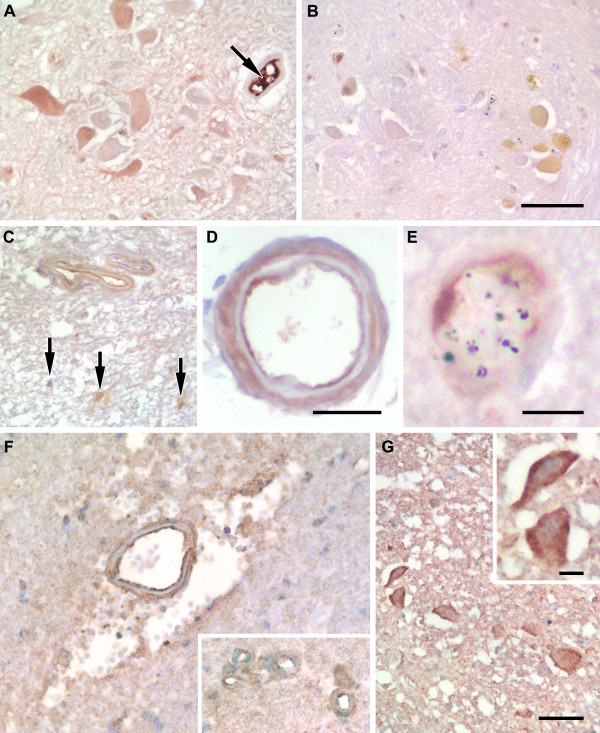
**Characteristic staining patterns for Epo and CD131 in the medulla of the brainstem**. **A**. Strong intravascular serum staining for erythropoietin (Epo, arrow). A range of staining intensities for Epo in the cytoplasm of neurons. **B**. Immunostaining for Epo in the nucleus and cytoplasm of neurons. Neurons and small vessels without Epo staining can be observed in this field. **C**. Two adjacent vessel showing strong endothelial and lighter pericyte/smooth muscle cell labelling for Epo. Glial cells with Epo labelling of processes are also shown (arrows). **D**. High power image of a pial vessel showing endothelial and pericyte/smooth muscle labelling. **E**. High power image of a parenchymal vessel with sequestered malaria parasites and Epo labelling of endothelial cells. **F**. Immunolabelling for CD131 on large and small (see insert) vessels. Labelling is found on both endothelial cells and pericytes/smooth muscle cells. The large vessel shows perivascular haemorrhage and oedema. **G**. Neurons from a control case with ischaemia showing immunolabelling for CD131. Staining is cytoplasmic with enhancement at the plasma membrane (see insert). Scale bars: A-C, F, G, 50 μm; D, 25 μm; E, 10 μm.

**Figure 2:**
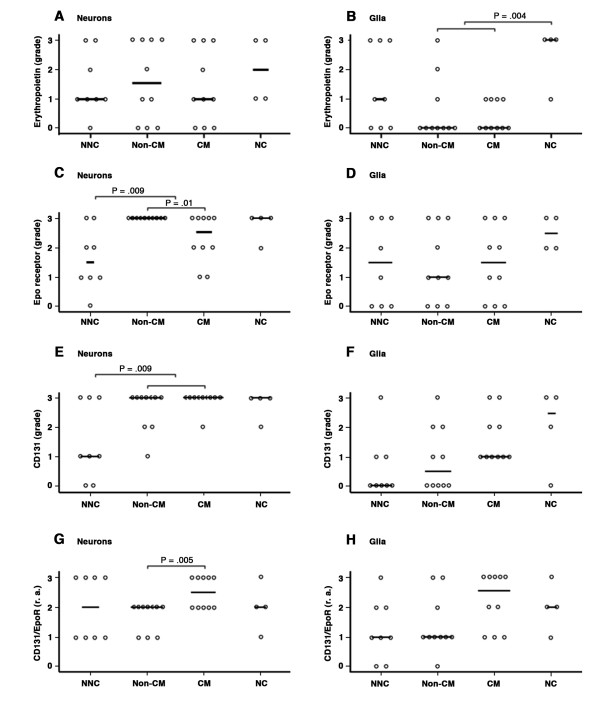
**Frequency of immunoreactivity for Epo, EpoR and CD131 on neurons and glia**. **A-F**. The degree of staining per tissue section with the various markers was semi-quantitated using the following grading scale: 0, no staining; 1, <1% cells staining; 2, 1%-10% of cells staining; 3, >10% cells staining. **G-H**. The relative abundance (r.a.) of CD131 to EpoR was determined for each brainstem section: 0, no staining for either CD131 or EpoR; 1, grade of immunolabelling for CD131 < EpoR; 2, grade of immunolabelling for CD131 = EpoR; 3, grade of immunolabelling for CD131 > EpoR. Each dot represents the results from a brainstem section of an individual patient. Results are divided into patient groups: NNC, non-neurological UK control; non-CM, non- cerebral malaria; CM, cerebral malaria; NC, neurological UK control (see Tables 1 & 2 for detailed patient descriptions).

Epo protein was expressed strongly in the cytoplasm and or nucleus of a range of cells in the severe malaria and control groups (Figures 1 &2A-B). The most striking staining was seen in neuronal cell bodies across all groups (Figures 1A-B &2A). Epo labelling of axons was only observed in one CM case and was not observed in non-CM sections. Epo labelling of glia was not a consistent feature of severe malaria (Figure 1C, arrows) but strong immunolabelling was found in the UK neurological control group (Figure 2B). The frequency of Epo labelling of glia was significantly lower in severe malaria cases compared with the neurological controls (*P *= .004). Endothelial labelling for Epo could be observed in 30% (6/20) of brainstem sections from severe malaria cases (Figure 1C-E), but could not be identified in the neurological controls. Intravascular serum staining for Epo was found in 75% (15/20) of severe malaria and 100% (4/4) of UK neurological control cases (Figure 1A, arrow) which would obscure positive endothelial labelling. Epo was also observed on pericytes/smooth muscle cells (Figure 1C-D) confirmed by smooth muscle actin immunostaining (data not shown).

#### EpoR

**Figure 3:**
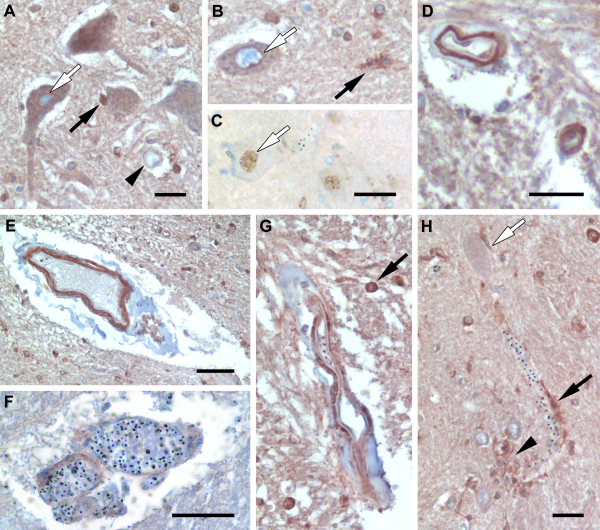
**Characteristic staining patterns for Epo receptor in the medulla of the brainstem**. **A-C**. Neurons staining for the erythropoietin receptor (EpoR). **A**. Cytoplasmic staining of neurons and perinuclear enhancement (white arrow). Nuclear and perinuclear staining of glia (filled arrow) and immunostaining of a small vessel (arrow head) can also be observed in this field. **B**. Cytoplasmic and strong perinuclear staining for EpoR in a neuron (white arrow) and cytoplasmic staining of microglia for EpoR (filled arrow). **C**. Nuclear staining for EpoR in a neuron. There is no cytoplasmic staining of neurons or vessels in this field. **D**. A small and medium sized vessel showing strong EpoR staining. **E-G**. Large vessels showing different patterns of EpoR immunolabelling. **E**. EpoR staining on the outer vessel structures and no staining on endothelial cells. **F**. Light EpoR staining associated with a vessel showing intravascular parasite burden. **G**. EpoR staining on endothelial cells and pericytes/smooth muscle cells. This image also shows nuclear staining in glia (filled arrow). **H**. A small vessel with intravascular parasites showing perivascular glial immunolabelling for EpoR (filled arrow). A perineuronal glia (white arrow) and a cluster of glia (arrow head) showing immunolabelling for EpoR. Scale bars: A, D, H, 25 μm; B-C, 25 μm; E-F, 50 μm.

In general, there was a greater frequency of cytoplasmic and/or nuclear EpoR labelling of cells within the brainstem sections compared with Epo (Figures 2A-D &3). The frequency of EpoR in neuronal cell bodies of severe malaria cases (Figure 3A-C) was greater than in non-neurological controls (*P *= .009, Figure 2C). EpoR labelling of axons was only observed in one CM section and was not observed in non-CM. There was a greater incidence of EpoR in neurons in the non-CM group compared with the CM group (*P *= .01). However, this was not the case for glia. Neurological controls showed consistently high levels of glial labelling for EpoR whereas the severe malaria and non-neurological controls showed a heterogeneous incidence of staining within each group (Figure 2D). EpoR immunolabelling was associated with vessels of varying calibre in 80% (16/20) of the severe malaria brainstem sections (Figure 3D-H). Labelling was associated with endothelial cells, pericytes/smooth muscle cells, perivascular glia (Figure 3H) of the BBB as well as intravascular monocytes. Of these cases, 35% (7/20) showed labelling of endothelial cells. There was no statistical difference between the frequency of EpoR labelling between vessels or endothelial cells and the different patient groups (*P *> .42).

#### CD131

CD131 showed staining of the same cellular elements as EpoR (Figure 1F-G). There was a greater frequency of labelling of neuronal cell bodies in severe malaria compared with non-neurological controls (*P *= .009, Figure 2E). CD131 labelling of axons was observed in sections from two non-CM cases. In line with the findings for EpoR, CD131 levels on glia were heterogeneous across all patient groups (Figure 2F). CD131 was found on the same vascular elements of small and larger vessels in severe malaria sections as EpoR, but the incidence was much lower (CD131: 30% (6/20) versus EpoR: 80% (16/20)). CD131 labelling of endothelial cells was observed in 25% (5/20) of cases (Figure 1F).

Double labelling for both EpoR and CD131 was not possible in this study so concordance of the incidence of marker expression was examined in serial sections. Although CD131 showed staining of the same cell subsets as EpoR concordance was not always observed. The strongest concordance occurred with neurons where 60% (12/20) of the severe malaria cases showed the same regional staining pattern and incidence of staining which was a score of 3 for both markers. Glial staining of EpoR and CD131 was heterogeneous across cases and discordant in incidence and location in serial sections with only 15% (3/20) of severe malaria cases showing a similar incidence of both markers and one case showing no staining for either marker. Vessels showed a predominance of EpoR labelling (see above), only 20% (4/20) of severe malaria cases showed the same incidence of both markers and 20% (4/20) of cases showed no staining for either marker. The relative abundance of EpoR to CD131 is described in section 6ii and Figure 2G-H.

### Relationship between Epo, EpoR and CD131 immunostaining, parasite load and cerebral sequestration

Among the severe malaria cases the mean admission peripheral parasite count was 270 500 per mm^3 ^(95% CI (lower bound - upper bound): 130 800 - 410 100). At the time of death the mean parasite load (% vessels sequestered) in the brainstem was 31.42% (95% CI: 13.71 - 49.13). There was a positive correlation between the frequency of EpoR on vessels (including endothelial cells, pericytes/smooth muscle cells and glial endfeet; see Figure 3) and admission parasite count (*P *= .01, r = .56). The median sequestration levels observed on sections with Epo, EpoR or CD131 immunolabelling on endothelial cells was greater than sections without marker labelling but this was not statistically significant (Epo: 5.00 [3.64-46.51] versus 53.0 [8.79-89.60]; EpoR: 4.00 [2.69-41.61] versus 57.5 [8.71-94.29]; CD131: 6.00 [9.30-48.30] versus 33.5 [0-115.01]; Median [95%CI]).

There was a strong correlation between the grade of EpoR associated with vessels and sequestration levels in the brainstem (*P *< .0001, r = .72). Sections with low sequestration load (<50% vessel sequestration) did not show any staining for CD131 on pericytes/smooth muscle cells but 43% (3/7) of sections with high sequestration showed CD131 in this cell type (*P *= .04). No staining of Epo or its receptors was seen in sequestered parasitized erythrocytes.

### Relationship between Epo, EpoR and CD131 immunostaining and vascular permeability and haemorrhage

There was heterogeneity in the amount of fibrinogen leakage in the brainstem of patients with severe malaria and controls (see Table 3). In general, median levels of fibrinogen leakage for the non-neurological control group were less than the severe malaria group and the median levels of fibrinogen leakage in the neurological UK controls were greater than the severe malaria group. However, due to the heterogeneity within groups this was not statistically significant. There were no statistically significant correlations between Epo, EpoR or CD131 and fibrinogen leakage. Haemorrhage, a consequence of frank vessel rupture, was semi-quantitated in the brainstem sections (see Table 4, [25]). There was a trend for a greater incidence of haemorrhage in sections without EpoR in glia compared with sections with EpoR in glia (*P *= .04).

**Table 3:** Fibrinogen leakage in the brainstem of severe malaria cases and UK controls

**Fibrinogen Leakage**	**Non-neurological UK controls**	**Non-CM**	**Cerebral malaria**	**Neurological controls**
High	13.29 [8.05-26.12]	32.22 [15.09-54.50]	18.97 [9.32-47.32]	54.39 [5.70-91.53]
Medium	18.27 [10.48-25.75]	21.47 [12.83-29.89]	22.60 [14.61-27.65]	16.19 [9.49-25.45]
Low	68.91 [48.83-80.77]	39.82 [23.82-63.87]	50.53 [30.48-70.61]	25.69 [0-74.57]

Geometric mean of fibrinogen load (%tissue section area) [95% confidence intervals]

**Table 4:** Summary of neuropathological features in the brainstem of fatal severe malaria cases

**Neuropathological feature**									
	**Haemorrhages** **(% severe cases, n = 20)**		**Astrogliosis** **(% severe cases, n = 19)**		**Swollen Axons** **(% severe cases, n = 20)**		**Chromatolysis** **(% severe cases, n = 20)**		
**Grade**	**NCM**	**CM**	**NCM**	**CM**	**NCM**	**CM**	No. centres effected*	**NCM**	**CM**
0	40	20	15.8	26.3	20	10	1	25	10
1	0	10	36.8	15.8	15	10	2	10	10
2	0	15	0	5.3	15	30	3	15	25
3	10	5	0	0	0	0	4	0	5

* All cases had at least 1 centre with chromatolytic neurons. Brainstem centres included: oculomotor, motor Vth, VIIth, vestibular, reticular formation and raphe, lateral cuneate, deep pontine and Xth [25].

### Relationship between Epo, EpoR and CD131 immunostaining and organ failure

Clinicopathological correlations were performed for the frequency of Epo, EpoR and CD131 immunolabelling and jaundice, anaemia, acute renal failure, hypoglycaemia, pulmonary oedema and haemodynamic shock. Cases with acute renal failure had a lower frequency of EpoR labelling of glia in the brainstem (*P *= .02). There were no statistically significant correlations.

### Relationship between Epo, EpoR and CD131 immunostaining and indicators of hypoxia

Among the severe malaria cases, the plasma and CSF lactate levels (6.1 ± 2.16 mmol/L and 6.4 ± 2.46 mmol/L, median ± SD, respectively) and plasma and CSF lactate pyruvate ratios (192.35 ± 187.15 and 42 ± 18.03, respectively) were greater than levels reported for normal adults (normal values: plasma lactate, 0.97 mmol/L; CSF lactate, 1.1-2.2 mmol/L; lactate pyruvate ratio 20:1 in normal biological fluids [34]). Arterial oxygen saturation was measured on admission using pulse oximetry. Median arterial oxygen saturation was 97% (range: 90.8 - 99.2%).

There was a strong negative correlation between arterial oxygen saturation measured on admission and EpoR labelling of glia (*P *= .001, r = -.82). Median admission arterial oxygen saturation was lower in sections with EpoR labelling of smooth muscle/pericytes (*P *= .01). There was no correlation between admission plasma lactate levels or the plasma or CSF lactate pyruvate ratio and the frequency of Epo, EpoR and CD131 immunostaining on neurons. There was a trend toward a positive correlation between CSF lactate levels and the frequency of Epo labelling of glia (*P *= .04, r = .58).

### Relationship between Epo, EpoR, CD131 and neuroprotection

#### (i) Axonal injury, astrogliosis and neuronal chromatolysis

A semi-quantitative assessment of swollen axons and astrogliosis and quantitation of the number of centres with chromatolytic neurons in the brainstem from formalin-fixed paraffin embedded sections from the same patients had been determined in a previous study [25]. Transverse sections were analysed between the midbrain and the pontomedulllary junction, stained with haematoxylin and eosin, solochrome-cyanin, and periodic acid-Schiff. A summary of the findings is shown in Table 4. There were no correlations between the frequency of Epo, EpoR or CD131 labelling in the medulla and the neuropathological features in the brainstem of the same cases with the exception of Epo labelling of neurons and astrogliosis (r = .85, *P *< .0001).

#### (ii) Relative abundance of CD131 to EpoR

In severe malaria, there was a greater relative abundance of CD131 compared to EpoR on neurons in CM patients compared with non-CM cases (*P *= .005; Figure 2G). There were no statistical correlations with glia, endothelial cells or vessels.

## Discussion

The aims of this study were to define the endogenous expression of Epo and its receptors in the brainstem of cases of fatal severe malaria and identify potential systemic and local regulatory factors. Whether different expression levels or differences in the abundance of receptor components could account for the extent of structural neuropathological changes was also investigated. The ultimate aim was to provide a scientific-based rationale for or against the use of Epo as a neuroprotective adjuvant treatment strategy in adults with severe malaria.

The frequency of cell-associated Epo labelling in the brainstem of severe malaria cases was not strikingly different from non-infectious, non-neurological fatal cases from the UK. However, Epo receptor labelling of neurons was more frequent in severe malaria compared with the non-neurological controls. Epo produced within the brain and systemically produced soluble Epo that accessed the brain could not be distinguished in this study. Recombinant human Epo (rhEpo) delivered systemically in rats is found associated with cerebral capillaries when examined 5 h later. At 17 h, pericapillary Epo is absent and, instead, localized to scattered neurons [35]. Epo in serum was observed in 75% of the severe malaria brainstem sections and would have had access to the brain either via a receptor-mediated transport mechanism in the cerebral endothelium or increased vascular permeability [13,20,35]. Epo expression tends to normalize shortly after the onset of hypoxia or traumatic brain injury [36] whereas EpoR can be induced for several days [37] which may explain the observed differences in the incidence of markers in the medulla of the malaria cases.

Correlation between the frequency of the markers on glia and vascular-associated cells was strongest with markers of cerebral hypoxia (CSF lactate) and systemic oxygen availability (arterial oxygen saturation), peripheral parasite load and sequestration. These findings are in agreement with the literature showing that Epo and EpoR are upregulated by hypoxia-inducible factors [15-18]. Epo has been reported to inhibit cerebral oedema [20,38,39], protect against vascular endothelial growth factor-induced permeability of the BBB, and restore junctional proteins [20]. There were no correlations between the frequency of Epo, EpoR or CD131 labelling and fibrinogen leakage. This supports our findings of multifactorial mechanisms leading to increased vascular permeability in severe malaria (Medana et al., personal communication).

The homodimeric EpoR receptor had been considered the sole receptor responsible for Epo functions. However, carbamylated Epo, a derivative of Epo, does not bind the homodimeric receptor yet has neuroprotective actions, facilitates clinical recovery in various animal models and appears to require the formation of a heteromeric receptor consisting of EpoR and CD131 [28,40]. Although no studies have directly shown a role for CD131 in binding Epo to the surface of any cell (reviewed in [41]), CD131 has been shown to physically and/or functionally associate with EpoR and coexpression of these receptors have been observed using immunohistochemistry in tissue sections from neurological disease models in animals [28,42,43]. It has even been suggested by some authors that a shift in abundance of hetero- to homodimeric receptors may divert Epo away from triggering potentially neuroprotective signals [3]. In this study there was a greater incidence of EpoR in neurons in non-CM cases compared with CM cases. However, there was a greater relative abundance of CD131 to EpoR in CM cases compared with non-CM cases. Taking all severe malaria cases together, the greatest concordance of EpoR and CD131 occurred with neurons (60% of cases). Although the relative contribution of neuroprotective Epo signalling in severe malaria is still not clear, there is no evidence to suggest that cerebral complications are due to a lack of CD131 availability and potential heteromeric receptor formation in neurons of the medulla. The incidence of EpoR and CD131 were discordant in glia and vessels with heterogeneous staining in the former and a predominance of EpoR in the latter. The way in which Epo exerts neuroprotective signalling in different cell types within the brain remains to be clarified.

Neurological complications reflect neuronal dysfunction. Previous studies in this group with severe malaria have shown no evidence for irreversible neuronal injury. However, there are neuronal stress responses, neuronal chromatolysis and axonal injury [25,26,31]. Chromatolysis was observed in at least one brainstem centre in all of the severe malaria patients analysed (n = 20, [25]), but endogenous Epo and receptor levels in the medulla did not reflect the extent of neuronal chromatolysis in the brainstem.

It has been shown previously that axonal injury was quantitatively greater in CM cases compared with severe malaria cases without neurological complications which may represent a final common pathway leading to neurological dysfunction in CM [26,44]. Swollen axons are commonly observed in these brainstem cases (Table 4). In the current study, Epo receptors were rarely observed on axons and Epo positive glia were not distributed around axonal fibre tracts. Two studies of traumatic brain injury (TBI) in mouse models have investigated the relationship between axonal injury and endogenous/supplemented Epo. In the first there was no significant difference between the amount of axonal injury in EpoR null mice (with endothelial EpoR restored) and wild type mice [37]. In the other model of TBI, mice were treated with recombinant human Epo (rhEpo). Similar amounts of axonal injury were observed in the treated and untreated groups during the acute phase of the degenerative process but axons were relatively better preserved in the treated group at later time points [45]. In this model, there is considerable neuronal damage and it could not be concluded whether the protective effect of Epo on axons is an epiphenomenon to the protection of neurons or a direct effect on axons. More recently, the possibility that Epo may amplify axonal injury in its early stages following TBI has been highlighted. The proposed mechanism suggests that Epo could increase axonal calcium levels as it has been shown to activate neural voltage-gated calcium channels [46]. Axonal injury in severe malaria is likely to involve increased levels of intracellular calcium reflected by increased levels of the calcium-activated cysteine protease, calpain [47].

Two mechanisms by which Epo is thought to provide cytoprotection is by increasing levels of heat shock protein 70 (HSP70) and by inhibition of caspase 3 [48,49]. HSP70 acts by binding to denatured proteins to restore their structure and function enabling the proper functioning of cells in a hostile environment. Caspase 3 is activated early in the apoptotic cascade, at a stage when commitment to loss of cell viability is made and is required for some hallmarks of apoptosis such as dismantling of the cell and the formation of apoptotic bodies (reviewed in [31]). In a previous study of these markers in brainstem sections a robust induction of heat shock protein 70 and minimal activated capsase 3 labelling of neurons was observed in severe malaria cases. Activated caspase 3 labelling of glia could not be distinguished from non-specific agonal levels observed in control groups [31]. Whether the regulation of HSP70 and caspase 3 is a consequence of Epo signalling or secondary to other preconditioning responses is unknown. However, it does raise the question of whether additional exogenous Epo would be of any benefit.

Cytoadherence of parasitized red blood cells to endothelial cells (EC) is central to the pathogenesis of cerebral and severe malaria [19,50]. Epo can contribute to neuroprotection through its actions on the endothelium such as EC survival, progenitor mobilization and angiogenesis and modulation of endothelial NO production (reviewed in [27]). NO is a potent vasodilator that is generated in endothelial cells from L-arginine by constitutively expressed endothelial NO synthase (eNOS). In this study, a greater incidence of EpoR was observed on cerebral vessels under conditions that would benefit from enhanced oxygen delivery. It can be envisaged that Epo induced eNOS could be protective in the context of severe malaria infection by preserving cerebral blood flow, facilitating effective collateral microcirculation, and anti-inflammatory effects. However, it has been recognized that severe malaria patients have reduced NO bioavailability due to hypoarginaemia and haemolysis [51]. Treatment of severe malaria cases with exogeneous Epo may, therefore, only have a limited effect on the vasculature in the absence of the eNOS signalling pathway irrespective of the presence of increased numbers of Epo receptors. In this study there was no correlation between the expression of Epo receptor components on vessels and markers of vascular injury such as plasma protein leakage and haemorrhage. It should also be considered that severe malaria patients with endothelial dysfunction caused by decreased NO bioavailability could be at a higher risk of adverse effects of exogenous Epo treatment such as increased blood viscosity, thrombosis and increased expression of the vasoconstrictor endothelin-1 (reviewed in [52]).

## Conclusion

The incidence of endogenous Epo in parenchymal brain cells did not greatly differ between severe malaria and non-neurological UK controls at the time of death. However, the incidence of Epo receptor component levels in neurons in severe malaria was greater than controls, suggesting increased receptivity to Epo. The relative contribution of Epo signalling through homodimeric or heteromeric Epo receptors is still not clear although there was no evidence to suggest that histological or neurological complications of severe malaria are due to a lack of CD131 availability and potential heteromeric receptor formation in neurons. It has been hypothesized that axonal injury may represent a final common pathway leading to neurological dysfunction in CM. There was no indication that axons would be directly receptive to Epo treatment since axons rarely expressed Epo receptors. Correlations were observed between the incidence of EpoR on vessels and markers of oxygen availability and sequestration. However, there were no correlations between the incidence of Epo or its receptors on vessels and haemorrhage or increased vascular permeability. Much of the evidence used to suggest Epo as an adjuvant in severe malaria is based on the mouse model of CM. Its mode of action in this model is the prevention of neuronal apoptosis and inflammatory responses, that are not characteristic features of CM in adults from south-east Asia. Data from other studies imply that pharmacological doses of Epo may have some value in ameliorating cerebral infarcts in large vessel territories, although these are infrequent in adults with CM. However this neuropathological study of endogeneous Epo and its receptors at the microvascular level does not provide a specific justification for the use of Epo as an adjuvant treatment in CM.

## Competing interests

The authors declare that they have no competing interests.

## Authors' contributions

IM was involved in the design of the study on the post-mortem tissues, carried out the immunostaining, together with GT assessed the immunolabelling at the microscope, performed the quantitative image analysis and statistics and wrote the manuscript. ND, TTH and NW were involved in the acquistion of clinical data and samples and the analysis and interpretation of the clinicopathological correlations, and revised the manuscript for important intellectual content. GT was involved in the design and coordination of the project and the analysis and interpretation of the clinicopathological correlations, assessed immunolabelling at the microscope, and revised the manuscript for important intellectual content. All authors read and approved the final version of the manuscript.
